# KI in der instrumentellen Ganganalyse: Herausforderungen und Lösungsansätze

**DOI:** 10.1007/s00132-025-04712-w

**Published:** 2025-09-02

**Authors:** Dominik Raab, Falko Heitzer, Christine Kocks, Wojciech Kowalczyk, Andrés Kecskeméthy, Marcus Jäger

**Affiliations:** 1 Lehrstuhl für Mechanik und Robotik, Universität Duisburg-Essen, Lotharstraße 1, 47057 Duisburg, Deutschland; 2 Lehrstuhl für Orthopädie und Unfallchirurgie, Universitätsklinikum Essen, Essen, Deutschland; 3 Klinik für Orthopädie, Unfall- und Wiederherstellungschirurgie, St. Marien-Hospital Mülheim, Mülheim an der Ruhr, Deutschland; 4 Lehrstuhl für Produktentstehungsprozesse und Datenmanagement, Universität Duisburg-Essen, Duisburg, Deutschland; 5 Klinik für Orthopädie, Unfall- und Wiederherstellungschirurgie, Philippus Stift, Essen, Deutschland

**Keywords:** Maschinelles Lernen, Gangstörungen, Muskuloskelettale Erkrankungen, Biomechanik, Motion Capture, Machine learning, Gait disorders, Musculoskeletal diseases, Biomechanics, Motion capture

## Abstract

**Hintergrund:**

Künstliche Intelligenz (KI) gilt als Schlüsseltechnologie zur Entlastung des Gesundheitssystems. Für die instrumentelle Ganganalyse, welche bislang durch aufwendige, manuelle Auswertungen von umfangreichen Patientendaten geprägt ist, versprechen KI-basierte Auswertungen für Orthopädie und Unfallchirurgie einen direkten und intuitiven Zugang zu klinisch relevanten Informationen.

**Ziel:**

Die spezifischen Herausforderungen, welche sich beim Einsatz von KI zur klinischen Auswertung ganganalytischer Messdaten ergeben, systematisch herauszuarbeiten und mögliche Lösungsansätze zu diskutieren.

**Methode:**

Zusammenführung einer systematischen Literaturrecherche zum Einsatz von KI in der Ganganalyse mit eigenem publiziertem Erfahrungswissen zur Anwendung von KI-Methoden in ganganalytischen Forschungsprojekten.

**Ergebnisse:**

Die KI-gestützte Verwertung von Ganganalysedaten ist durch sechs zentrale Herausforderungen gekennzeichnet. Die Hauptherausforderung besteht darin, dass KI-Methoden am besten funktionieren, wenn umfangreiche Trainingsdaten, wenig Einflussgrößen und eindeutige Zielgrößen vorliegen, während instrumentelle Ganganalyse durch gegenläufige Rahmenbedingungen geprägt ist (wenig Trainingsdaten, mannigfaltige Einflussgrößen, unscharfe Zielgrößen). Zur Kompensation dieser ungünstigen Voraussetzungen wird ein Katalog möglicher Lösungsansätze skizziert, in dessen Zentrum eine möglichst umfassende Einbindung von klinischem Expertenwissen in die KI-Entwicklung steht.

**Diskussion:**

KI bietet ein großes Potenzial zur Verbesserung des klinischen Zugangs zur instrumentellen Ganganalyse. Die Schwelle für den klinischen Einsatz von KI ist jedoch noch hoch, da es bislang an Leitlinien zur Begegnung von anwendungsspezifischen Herausforderungen sowie einer zielgerichteten klinischen Integration mangelt.

**Graphic abstract:**

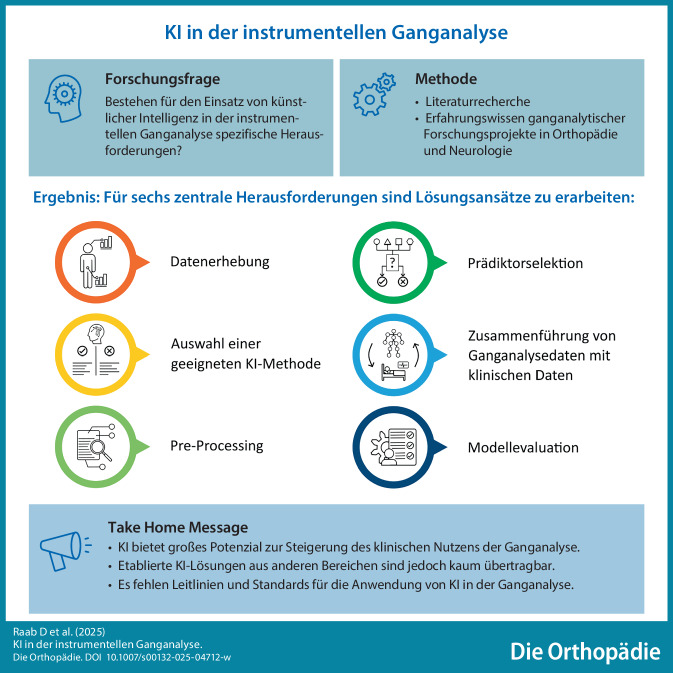

## Abstract (en)

**Background:**

Artificial intelligence (AI) is considered a key technology for alleviating the burden on the healthcare system. For instrumental gait analysis, AI-based evaluations promise a direct and intuitive access to clinically relevant information in orthopaedics and trauma surgery, avoiding the challenging and time-consuming manual evaluations of large amounts of patient data.

**Objective:**

The objective of this work is to investigate the specific challenges and limitations of using AI for clinical evaluation of gait analysis data and to propose effective solutions to address these limitations.

**Method:**

This work combines a systematic literature review on AI in gait analysis with practical experiences from applications of AI in the authors′ own published research projects.

**Results:**

Six key challenges have been identified. While AI methods work best when extensive training data, a limited number of influencing factors, and a clearly defined target variable are available, instrumental gait analysis is characterised by opposite conditions (little training data, multiple influencing factors, and fuzzy target variables). To address these contradicting characteristics, a catalogue of possible solution approaches focusing on integrating clinical expert knowledge into AI development and operation is outlined.

**Conclusion:**

It is shown that AI offers significant potential for improving the efficiency and quality of gait data exploitation. However, current AI approaches from other fields are only partially transferable to gait analysis due to insufficient fitting. By addressing the specific challenges for AI in gait analysis, it can be expected that specialized procedures and best practices can be developed, which will boost AI assistance in IGA clinical evaluation.

Künstliche Intelligenz (KI) gilt als Schlüsseltechnologie zur Entlastung und Effizienzsteigerung des Gesundheitssystems. Vom Einsatz von KI-Methoden können insbesondere klinische Anwendungsgebiete profitieren, die bislang durch aufwendige, expertenbasierte Auswertungen von komplexen und vielschichtigen Patientendaten geprägt sind. Bei der Ausarbeitung von KI-basierten Unterstützungssystemen ist jedoch zu bedenken, dass jedes klinische Anwendungsgebiet durch spezifische Herausforderungen gekennzeichnet ist, für die anwendungsorientierte Lösungsstrategien zu entwickeln sind.

## Hintergrund und Fragestellung

Die instrumentelle Ganganalyse (IGA, OPS-Code: 1‑798) ist ein anerkanntes diagnostisches Verfahren zur physiologischen Funktionstestung und etabliert sich in der klinischen Versorgung von muskuloskelettalen und neurologischen Erkrankungen des Bewegungsapparates zunehmend als wertvolle Ergänzung zur konventionellen Diagnostik. IGA bietet mittels Kombination eines Motion-Capture-Systems zur Bewegungserfassung mit Begleitsystemen eine hochauflösende, dreidimensionale Vermessung von Struktur, Bewegung und Belastung des Bewegungsapparates. Dies ermöglicht eine differenzierte Beurteilung und Dokumentation der Funktionalität des Bewegungsapparates anhand von quantitativen Kriterien und bietet somit ein nützliches Werkzeug für die Diagnostik von komplexen Bewegungsstörungen von Einzelpersonen sowie zur Durchführung von Quer- und Längsschnittstudien [9].

**Abb. 1:**
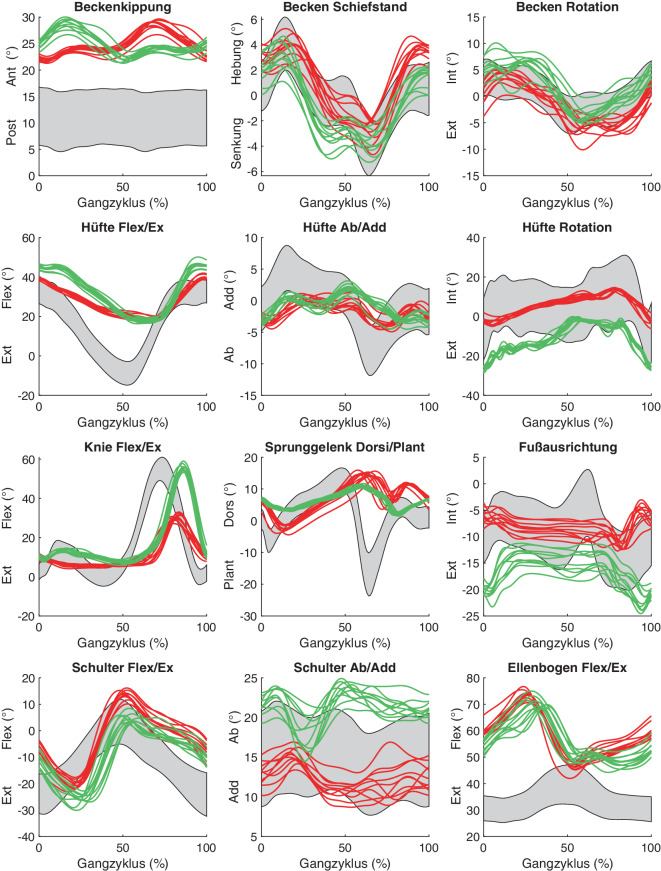
Auszug aus den Ganganalysedaten eines Patienten mit linksseitiger, schmerzhafter Pathologie am Kniegelenk; *rot* linke Gangzyklen, *grün* rechte Gangzyklen, *grau* Durchschnittswerte gesundes Vergleichskollektiv

Trotz dieser Vorteile hat sich IGA im Fach Orthopädie und Unfallchirurgie noch nicht flächendeckend etabliert. Dies ist vorwiegend begründet im erforderlichen Expertenwissen für die Interpretation der Messdaten und dem damit verbundenen Zeit- und Personalaufwand. Abb. 1 veranschaulicht die Komplexität und Vielschichtigkeit von Ganganalysedaten anhand eines Patienten mit linksseitiger Pathologie am Kniegelenk. Die dargestellten Graphen bilden jeweils den Zeitverlauf eines Körper- oder Gelenkwinkels für mehrere, auf 100 % normalisierte Gangzyklen der linken und rechten Körperseite ab (rote bzw. grüne Kurven) und setzen diese in Relation zu den Durchschnittswerten eines gesunden Vergleichskollektivs (grauer Schlauch). Die IGA-Daten werden meist komplettiert durch analoge Graphen zur Darstellung von Gelenkkräften und -momenten sowie Gangparametern zur Beschreibung von funktionalen Aspekten der Gangbewegung.

Die klinische Verwertung dieser Daten ist aus den folgenden Gründen eine Herausforderung:

- Die erhobenen Daten sind mannigfaltig in Art und Umfang. Für ihre Interpretation ist es daher unerlässlich zu erkennen, welche Informationen für die vorliegende Fragestellung relevant sind.
- Die Daten bestehen größtenteils aus Kurvenverläufen. Diese sind aufwendig zu interpretieren, da jeder Verlauf spezifische, befundungsrelevante Charakteristika aufweist.
- Es bestehen komplexe, nichtlineare Abhängigkeiten zwischen den erhobenen Daten. Eine Pathologie an einem Hüftgelenk kann beispielsweise aufgrund von Kompensationsmechanismen auffällige Winkelverläufe in Knie- und Sprunggelenk verursachen. Die einzelnen Informationen der IGA sind daher zu einem ganzheitlichen Gesamtbild zusammenzufügen und müssen mitunter durch weitere Daten ergänzt werden (Anamnese, Labordiagnostik, bildgebende Verfahren).
- Die Daten verteilen sich auf mehrere Gangzyklen, deren Kurvenverläufe oftmals deutliche Abweichungen in Absolutwerten und Form aufweisen (vgl. Streuung in Abb. 1). Durch diese inhärente Unschärfe ist die Beurteilung des charakteristischen Gangbildes einer Person und die Deutung von zugrundeliegenden Pathomechanismen schwierig.

Aus diesen Gründen ist für die klinische Interpretation von IGA-Daten ein sehr großes Maß an Erfahrung erforderlich. Die Technologie der Künstlichen Intelligenz (KI) verspricht an dieser Stelle Abhilfe. Unter diesem Begriff werden eine Vielzahl von Methoden des maschinellen Lernens zusammengefasst, welche Muster und Zusammenhänge innerhalb von vielschichtigen Datensätzen automatisiert erkennen können. Dies ermöglicht es, Lösungen für spezifische, klar definierte Aufgaben zu erlernen und eine effiziente, vollautomatisierte Verarbeitung von Datensätzen, die für eine manuelle Auswertung zu groß und/oder zu unübersichtlich sind, umzusetzen. So werden KI-Methoden in anderen Fachdisziplinen der Medizin bereits sehr erfolgreich eingesetzt, um in der Radiologie die Erkennung von Arealen mit veränderten Gewebestrukturen zu unterstützen [7, Kap. 22.4] oder in der Medikamentenentwicklung die Wirkung von Inhaltsstoffen vorherzusagen [7, Kap. 6]. Auch für die Verwertung der Messdaten der IGA bietet KI großes Potenzial. KI-Lösungen aus anderen Bereichen sind allerdings nur sehr eingeschränkt auf die IGA übertragbar, weil Strukturen und Umfänge von IGA-Daten nicht typischen KI-Anwendungen entsprechen. Für den erfolgreichen Einsatz von KI in der IGA sind daher spezifische Lösungsansätze erforderlich. In diesem Beitrag werden hierzu die wesentlichen Herausforderungen systematisch herausgearbeitet und mögliche Lösungsansätze vorgestellt.

## Methode

**Abb. 2:**
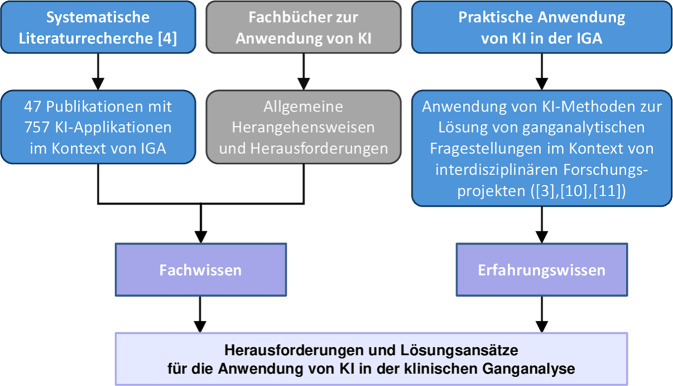
Methode; *IGA* instrumentelle Ganganalyse, *KI* Künstliche Intelligenz

Die Forschungsfrage, welche spezifischen Herausforderungen für den Einsatz von KI in der IGA bestehen, wurde mittels eines explorativen Lösungsansatzes untersucht, der auf einer Fusion von Fachwissen und Erfahrungswissen basiert (Abb. 2).

### Fachwissen

In einer systematischen Literaturrecherche in den Datenbanken PubMed, Embase und Google Scholar wurden 47 Übersichtspublikationen identifiziert, welche anhand von 757 Anwendungen von KI-Methoden in der IGA einen repräsentativen Überblick über das Themenfeld bieten [4]. Die Volltexte dieser Publikationen sowie eine Auswahl von allgemeinen Fachbüchern zur praktischen Anwendung von KI [6, 7, 15] wurden systematisch auf relevante Informationen zur Forschungsfrage ausgewertet. Die für die Literaturrecherche verwendete Suchanfrage lautete: „(artificial intelligence OR computational intelligence OR machine learning OR deep learning) AND (instrumental motion analysis OR instrumental gait analysis OR smart gait OR body modeling OR plug-in gait mode)“.

### Erfahrungswissen

Neben theoretischem Fachwissen aus Literaturquellen sind Erkenntnisse aus der praktischen Anwendung von KI-Methoden ein integraler Bestandteil der verfolgten Methodik. Im Kontext der interdisziplinären EFRE-Forschungsprojekte ReHabX [3], RehaBoard [10] und RehaToGo [11] konnten die Autoren dieses Beitrages eine breite Palette an KI-Methoden zur Lösung von vielfältigen ganganalytischen Fragestellungen einsetzen und somit eine probate Wissensbasis zum evidenzbasierten Einsatz von KI in der IGA aufbauen. Im Rahmen der genannten Projekte wurden insgesamt 560 Personen ganganalytisch vermessen und eine Vielzahl klinischer Begleitdaten erhoben. Die Patientenkohorte stammte nahezu im gleichen Anteil aus den Fachbereichen Orthopädie (40 %) und Neurologie (48 %), sodass die beiden wichtigsten Anwendungsfelder der IGA gleichermaßen repräsentiert sind. Ergänzt wurde der Datensatz durch ein Vergleichskollektiv gesunder Personen (12 %). Weiterhin wurden 110 der neurologischen Patienten in einem Expertenboard besprochen und deren disziplinübergreifende Befunde abgestimmt. Messtechnik, Ein- und Ausschlusskriterien, klinische Begleitdaten und angewandte KI-Methoden wurden jeweils in Abhängigkeit von den vorliegenden klinischen Fragestellungen gewählt. Nähere Details sind den zugehörigen Publikationen zu entnehmen [3, 10, 11, 16].

## Ergebnisse

**Abb. 3:**
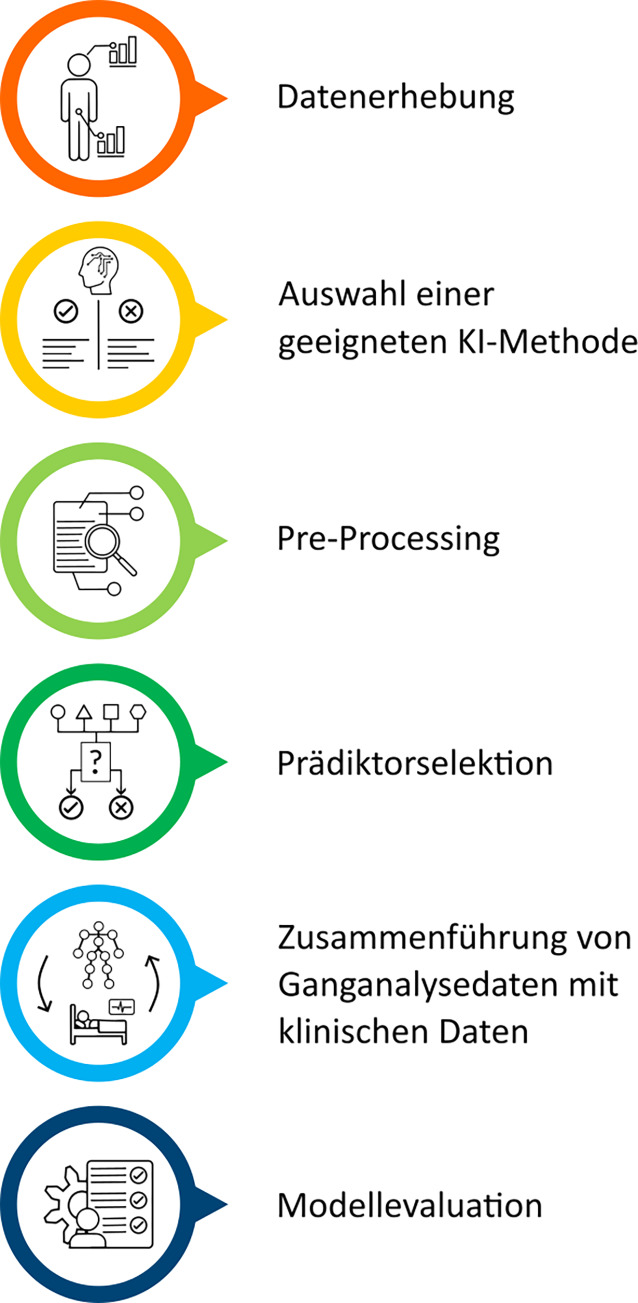
Übersicht Herausforderungen

Die Tab. 1 zeigt eine Übersicht darüber, wofür KI in der IGA bislang eingesetzt wird. Mit 88 % dient die überwiegende Mehrheit der IGA-Publikationen mit KI-Bezug bislang der Lösung von Klassifikations- und Regressionsproblemen. KI kommt somit vorwiegend zum Einsatz, um kategorielle Merkmale wie Gruppeneinteilungen vorherzusagen (67 %) oder metrische Merkmale wie Scores oder Kennzahlen zu prognostizieren (21 %). Anwendungen wie Dimensionsreduktion (5 %), Cluster-Bildung (2 %) und strukturierte Vorhersage (2 %) nehmen hingegen nur einen geringen Stellenwert ein. Die verwendeten KI-Methoden konzentrieren sich hauptsächlich auf künstliche neuronale Netze (36,3 %), entscheidungsbaumbasierte Verfahren (21,5 %) und Diskriminanzmethoden (20,3 %). Der Anteil aller anderen KI-Methoden liegt jeweils bei ≤ 1,7 %. Die auf Grundlage der Fachliteratur sowie dem vorliegenden Erfahrungswissen herausgearbeiteten Herausforderungen sind in Abb. 3 dargestellt und werden nachfolgend einzeln erläutert.

**Tab. 1:** Wesentliche KI-Anwendungen in der IGA (Prozentangaben aus [4])

Anwendungen*		Ziel	Verwendete KI-Methoden* (Auszug)
*Klassifikation *(67 %)	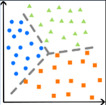	Vorhersage kategorieller Merkmale, z. B. Gruppeneinteilungen [8], Ereigniserkennung [2]	Künstliche neuronale Netze (28,5 %) Diskriminanzmethoden (20,3 %) Entscheidungsbäume (14,1 %)
*Regression *(21 %)	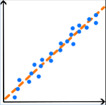	Prognose metrischer Merkmale, z. B. klinische Scores und Kennzahlen [11]	Künstliche neuronale Netze (7,8 %) Entscheidungsbäume (7,4 %) Lineare Regression (< 1 %)
*Dimensionsreduktion *(5 %)	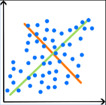	Zusammenfassung von unübersichtlichen Daten ohne wesentlichen Informationsverlust, z. B. Hauptkomponenten oder Singulärwerte [1]	Lineare Diskriminanzanalyse (1,7 %) Hauptkomponentenanalyse/Singulärwertzerlegung (1,7 %)
*Clusterbildung *(2 %)	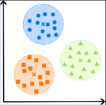	Zusammenfassung von gleichartigen Fällen in natürliche, trennscharfe Gruppen, z. B. Phänotypen [17]	„Self-organizing maps“ (< 1 %) „k-means clustering“ (< 1 %)
*Strukturierte Vorhersage *(2 %)	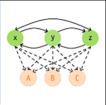	Vorhersage von komplexen Beziehungen zwischen mehreren Merkmalen, z. B. Abhängigkeit zwischen Beschleunigungsdaten und Bewegungsereignissen [12]	Hidden-Markov-Modelle (< 1 %), Bayes-Netze (< 1 %)

* Prozentangabe = Anteil der jeweiligen Anwendung bzw. KI-Methode an den 757 untersuchten Anwendungen von KI-Methoden; 17 Anwendungen (3 %) sind der Gruppe „Sonstige Aufgaben“ zuzuordnen, welche keine charakteristischen Eigenschaften aufweist; *KI* Künstliche Intelligenz

### Datenerhebung

Die Qualität der Daten, mit denen eine KI trainiert wird, ist ausschlaggebend für die Leistungsfähigkeit des KI-Modells und maßgeblich dafür, dass es für neue unbekannte Daten verlässliche Ergebnisse liefern kann. Daher ist es essenziell, der KI für die jeweilige Fragestellung sämtliche relevanten Informationen in geeigneter Menge und Verteilung bereitzustellen. Um Muster und Zusammenhänge reliabel erkennen zu können, benötigt eine KI idealerweise

- unverzerrte Trainings- und Testdaten, die das gesamte Spektrum relevanter Eigenschaften für die vorliegende Fragestellung repräsentativ und lückenlos abdecken,
- eine gleichmäßige Verteilung aller für die untersuchte Fragestellung relevanten Kombinationen von Eigenschaften,
- Begleitinformationen zur Kontextinterpretation,
- Vergleichsdaten zur Abgrenzung ähnlicher Muster (z. B. Patienten mit anderen Erkrankungen).

Für klinische Anwendungen am Stütz- und Bewegungsorgan bildet die Zusammenstellung von KI-geeigneten Datensätzen eine große Herausforderung. Dies ist bei prospektiven Datenerhebungen vorwiegend im großen Zeit- und Personalaufwand begründet, der mit der Durchführung von IGA-Studien einhergeht, sowie mit der Schwierigkeit, die benötigte Anzahl an Studienteilnehmern zu rekrutieren, welche die zuvor genannten Kriterien abdecken. Die retrospektive Zusammenstellung von Datensätzen ist in der IGA ebenfalls beschränkt, weil sie in den meisten Einrichtungen lediglich für sehr spezielle Fragestellungen zur Anwendung kommt, sodass einzelne Labore in der Regel nur über begrenztes Datenmaterial verfügen. Die Fusion der Daten mehrerer Labore scheitert hingegen oft an unterschiedlichen Ausführungen der IGA oder am Datenschutz bzw. an Zweckbindung der Daten.

### Auswahl einer geeigneten KI-Methode

Für die Auswahl einer geeigneten KI-Methode für Bewegungsanalysen des Stütz- und Bewegungsorgans steht eine Vielzahl von Verfahren mit unterschiedlichen Stärken, Schwächen und Anforderungen zur Verfügung. Eine zielgerichtete Auswahl der für den Anwendungsfall geeigneten KI-Methode erfordert ein tiefes Verständnis für

- die zugrunde liegende klinische Fragestellung,
- die Anforderungen an die KI-basierte Lösung (z. B. Ergebnisgüte, Komplexität, Erklärbarkeit),
- die Eigenschaften der vorliegenden Daten,
- die verfügbaren Ressourcen (Rechenleistung, Software-Lizenzen, humane Ressourcen),
- die Funktionsweise potenziell geeigneter KI-Methoden.

Die wesentliche Herausforderung in diesem Kontext liegt in der Notwendigkeit, tiefes klinisches Fachwissen mit datenwissenschaftlicher Kompetenz zu vereinen. In unserer Arbeitsgruppe arbeiten Ingenieure, Orthopäden, Unfallchirurgen, Neurologen, Physiotherapeuten und Orthopädietechniker seit über 10 Jahren kontinuierlich zusammen. Die Erfahrung zeigt, dass die Etablierung einer passenden Arbeitsgruppe stets hohe Anforderungen an eine fachdisziplinübergreifende Zusammenarbeit stellt.

### Pre-Processing

Pre-Processing (engl. Vorverarbeitung) umfasst eine Vielzahl von Arbeitsschritten, um Rohdaten in eine Form zu überführen, die sich für die weitere Verarbeitung mit KI-Methoden eignet. „Nicht selten entfällt auf das Pre-Processing 80 % der gesamten Arbeit an einem Projekt“ [15, S. 10]. Übliche Tätigkeiten des Pre-Processings umfassen:

- Datenkontrolle und -bereinigung: Identifikation fehlerhafter oder fehlender Werte sowie Bereinigung durch Korrektur bzw. Approximation von Werten oder Entfernung der fehlerbehafteten Fälle.
- Numerisches Encoding: Umwandlung kategorieller Merkmale (Eigenschaften, die nicht quantitativ sind, sondern qualitativ vorliegen wie z. B. Geschlecht, betroffene Körperseite) in numerische Werte als Vorbereitung für die Weiterverarbeitung mit Methoden, die ausschließlich numerische Eingangsdaten verarbeiten können.
- Skalierung der Daten: Vereinheitlichung des Wertebereichs, sodass alle Merkmale auf einer vergleichbaren Skala vorliegen.
- Feature-Engineering: Überführung der Rohdaten in Merkmale, welche spezifische, für die vorliegende Fragestellung relevante Eigenschaften quantifizieren. Dazu werden neuartige Merkmale entwickelt, indem Rohdaten transformiert oder mehrere Rohdaten kombiniert werden [6, S. 83].
- Splitting: Aufteilen der Daten in Teildatensätze für Modelltraining, -testung und ggf. -validierung.

Im Kontext der IGA ist zusätzlich zu berücksichtigen, dass pro Person typischerweise mehrere Gangzyklen vorliegen, deren Anzahl individuell variiert. Während des Pre-Processings ist daher zu entscheiden, welche Gangzyklen bei der Auswertung berücksichtigt werden. Die nachfolgende Aufzählung schlüsselt etablierte Methoden zum Pre-Processing von Gangzyklen auf und gibt an, wieviel Prozent der von [4] identifizierten Anwendungen auf die jeweilige Methode zurückgreifen:

- Verwendung eines einzelnen Gangzyklus pro Person (20 %). Dabei erfolgt die Auswahl des verwendeten Gangzyklus idealerweise mittels einer Methode zur Bestimmung des repräsentativen Gangzyklus, wie z. B. [14].
- Verwendung einer einheitlichen Anzahl an mehreren Gangzyklen pro Person (21 %).
- Verwendung aller vorhandenen Gangzyklen und Kompensation von unterschiedlichen Anzahlen an Gangzyklen pro Person mit Gewichtungsfaktoren auf Ebene der aus den Gangzyklen extrahierten Kenngrößen, z. B. per Mittelwertbildung (18 %).
- Verwendung aller vorhandenen Gangzyklen ohne Kompensation der unterschiedlichen Anzahl an Gangzyklen pro Person (12 %).

Bei 29 % der Anwendungen war der beschriebenen Methodik keine eindeutige Angabe zur Verarbeitung der Gangzyklen zu entnehmen.

### Prädiktorselektion

Um zu vermeiden, dass ein KI-Modell zu sehr auf Besonderheiten des Trainingsdatensatzes angepasst ist und dadurch schlecht auf neue Daten generalisiert, ist es sinnvoll, die Anzahl verwendeter Eingangsgrößen (sog. Prädiktoren) zu begrenzen. Die Auswahl und Anzahl der eingebundenen Prädiktoren beeinflussen maßgeblich die Güte und Erklärbarkeit des entwickelten Modells. In der IGA sind selten sämtliche relevante Einflussgrößen bekannt, die für eine rein sachlogische Prädiktorselektion erforderlich wären. Für eine datengetriebene Selektion sind die Voraussetzungen ebenfalls ungünstig, da einer geringen Fallzahl ein großer Pool an potenziellen Merkmalen mit komplexen, nichtlinearen Abhängigkeiten gegenübersteht. Dadurch ist die Auswahl der Prädiktoren stark von der Zusammenstellung der Trainingsdaten abhängig, sodass jede Variation der Trainingsdaten zu komplett anderen Modellen führen kann. Dies widerspricht dem Ziel, ein robustes und generalisierbares Modell zu entwickeln. Für die meisten klinischen Fragestellungen der IGA besteht somit die wesentliche Herausforderung darin, einen zielführenden und reproduzierbaren Ansatz zur Prädiktorselektion zu etablieren. Von den 47 gesichteten Übersichtspublikationen berücksichtigen 29 (62 %) diese Thematik.

### Zusammenführung von Ganganalysedaten mit klinischen Daten

Bei der Zusammenführung von Ganganalysedaten mit klinischen Daten ist folgendes zu berücksichtigen:

- Bei der Verwendung mehrerer Gangzyklen pro Person variieren die ganganalytischen Messwerte zwischen den einzelnen Gangzyklen, während die klinischen Daten der Personen konstant bleiben.
- Ärztliche Diagnosen und Maßnahmenempfehlungen sowie viele klinische Scores sind keine objektiven Messgrößen, sondern subjektiv geprägte Einschätzungen, die auf der Grundlage von fachlicher Expertise und gegebenenfalls auch Patientenauskünften getroffen werden. Somit kann nicht gewährleistet werden, dass eine wiederholte Erhebung der Daten zu identischen Ergebnissen unabhängig von der begutachtenden Person führt.

Diese Ausgangslage erschwert es, mit einer begrenzten Anzahl an Trainingsdaten verlässliche, allgemeingültige Zusammenhänge zwischen klinischen Informationen und Ganganalysedaten abzubilden. Dieser Aspekt ist für die Forschungsfragen von 62 % der gesichteten Übersichtspublikationen relevant.

### Modellevaluation

Um zu beurteilen, inwieweit ein erstelltes KI-Modell zur Lösung der vorliegenden Aufgabenstellung geeignet ist, bedarf es einer ausführlichen Evaluation. Wesentliche Aspekte dabei sind:

- Die Genauigkeit des Modells, welche durch Maßzahlen der Modellgüte quantifiziert wird.
- Die Fähigkeit des Modells, von Trainingsdaten auf unbekannte Daten generalisieren zu können, welche durch Methoden der Kreuzvalidierung approximiert werden kann.
- Die Anzahl, Art und Schwere auftretender Modellfehler.
- Die Auswahl von Merkmalen als Prädiktoren.
- Die Nachvollziehbarkeit der Entscheidungsfindung des KI-Modells. Bei Whitebox-Modellen, wie Entscheidungsbäumen, sind die Beziehungen zwischen Ein- und Ausgangsgrößen nachvollziehbar, sodass die Ergebnisfindung überprüfbar ist. Blackbox-Modelle, wie künstliche Neuronale Netzwerke, sind hingegen durch die komplexen und intransparenten Beziehungen zwischen den Ein- und Ausgangsgrößen gekennzeichnet. Dabei kann lediglich das Modellverhalten mit weiterführenden Methoden analysiert werden (z. B. SHAP [8]).

Die Eignung eines KI-Modells zur Lösung einer vorliegenden Aufgabenstellung zu bewerten, ist ein komplexes Unterfangen, weil die zuvor genannten Gesichtspunkte einzeln zu evaluieren und gegeneinander abzuwägen sind. Zudem existieren zahlreiche Bewertungsmetriken, die jeweils unterschiedliche Schwerpunkte setzen, sodass eine objektiv vergleichbare Bewertung so nicht möglich ist. Gerade bei klinischen Fragestellungen ist die Erklärbarkeit von KI-basierten Entscheidungen ein zentrales Kriterium für die Akzeptanz von KI im medizinischen Umfeld [7, Kap. 35]. Jedoch kann bei 64 % der von [4] identifizierten Anwendungen aufgrund der Verwendung von Blackbox-Modellen die Nachvollziehbarkeit von Modellentscheidungen nicht vollumfänglich gewährleistet werden.

## Diskussion von Lösungsansätzen

### Datenerhebung

Zeit- und Kostenrestriktionen sowie Verfüg- und Belastbarkeit von Patienten und medizinischem Fachpersonal setzen dem Umfang der prospektiven Erhebung von IGA-Daten natürliche Grenzen. Mögliche Ansätze zur Erzielung umfangreicher Datensätzen sind:

- Die Reduzierung des Aufwandes für die Datenerhebung durch Abkehr von der zeit- und personalaufwendigen laborbasierten Bewegungsanalyse hin zur Verwendung von einfachen, tragbaren Sensoren (sog. Wearables) [13]. Dabei ist jedoch zu berücksichtigen, dass der Einsatz von Wearables mit einer deutlichen Reduktion von Breite, Auflösung und Qualität der Messdaten einhergeht, sodass eine umfassende Abwägung erforderlich ist, inwieweit verfügbare Wearables zur Lösung einer spezifischen Fragestellung geeignet sind.
- Etablierung von Standards für Qualität und Umfang der Datenerhebung für IGA einschließlich sinnvoller Metadaten, hilfreicher klinischer Begleitdaten sowie erforderlicher Einwilligungen, um perspektivisch die Etablierung von großen, laborübergreifenden Datensätzen für KI-Entwicklungen zu ermöglichen. Ein umfassender Standardisierungsvorschlag für Bewegungslabore wurde 2025 von der GAMMA publiziert [5].

### Auswahl einer geeigneten KI-Methode

Eine probate Lösung zur Auswahl einer passenden KI-Methode für die vereinfachte Auswertung von IGA-Daten ist eine Best-Practice-Übersicht, welche für typische Anwendungen der IGA geeignete KI-Methoden nennt und deren Vor- und Nachteile gegenüberstellt. Aufgrund der Fülle potenziell relevanter KI-Methoden und der Breite möglicher IGA-Anwendungen stellt die Erarbeitung von entsprechenden Leitlinien jedoch ein umfangreiches Unterfangen dar, für das idealerweise ein interdisziplinäres Team aus den Bereichen IGA und Datenanalyse gebildet werden sollte.

### Pre-Processing Ganganalysedaten und Prädiktorselektion

Um trotz weniger Trainingsdaten, vielschichtigen Eingangsdaten und unscharfen klinischen Zielgrößen effektive KI-Anwendungen für die Bewegungsanalyse zu entwickeln, ist es essenziell, den KI-Algorithmen die für die Problemlösung erforderlichen Eigenschaften in einer lösungsorientierten Form zur Verfügung zu stellen. Aus Sicht der Autoren liegt daher der zentrale Lösungsansatz für die erfolgreiche Anwendung von KI in der IGA in der Entwicklung spezifischer Merkmale zur Abbildung von problemrelevanten Eigenschaften im Rahmen des Pre-Processings sowie in der sachlogischen Vorauswahl an lösungsrelevanten Merkmalen im Kontext der Prädiktorselektion. Auf diese Weise können ungünstige Rahmenbedingungen durch Einbringung von Fachwissen kompensiert werden. Für die erfolgreiche Bewältigung dieser Herausforderung ist eine enge, interdisziplinäre Zusammenarbeit zwischen klinischen Experten, IGA-Experten und Fachleuten der Datenanalyse von essenzieller Bedeutung.

### Zusammenführung von Ganganalysedaten mit klinischen Daten

Um die natürliche Unschärfe klinischer Zielgrößen abzufedern, sollte als Zielgröße für ein KI-Training nicht die Einschätzung einer Einzelperson dienen, sondern immer das Mehrheitsvotum einer repräsentativen Auswahl klinischer Experten verwendet werden. Dies ermöglicht zudem die Güte des KI-Modells im Kontext der Interrater-Reliabilität der Experten zu beurteilen.

### Modellevaluation

Eine umfassende inhaltliche Modellevaluation des KI-Modells erfordert die Beschränkung auf eine überschaubare Anzahl klinisch interpretierbarer Merkmale („Features“). Dies unterstreicht die Bedeutung der Entwicklung geeigneter und interpretierbarer Features im Rahmen eines gezielten Pre-Processings sowie einer wohldurchdachten Prädiktorselektion wesentlicher Features. Weiterhin sollten bevorzugt Whitebox-Modelle als verwendete KI-Methoden ausgewählt werden, sodass der Weg der Entscheidungsfindung überprüfbar ist. Bei Einsatz von Blackbox-Modellen ist hingegen stets eine Evaluation des Modellverhaltens mit weiterführenden erklärenden Methoden erforderlich.

## Resümee

KI bietet großes Potenzial zur Automatisierung und Qualitätssteigerung der klinischen Anwendung von IGA und ist in Kombination mit einer rasanten technischen Entwicklung im Bereich von Wearables der Schlüssel, um IGA von einer expertenbasierten Technologie für ausgewählte klinische Anwendungsfälle zu einer Standarddiagnostik für Bewegungsstörungen weiterzuentwickeln. Im Vergleich zu anderen Fachdisziplinen steckt KI in der Ganganalyse jedoch noch in den Kinderschuhen. Dies ist vorwiegend darauf zurückzuführen, dass für IGA-spezifische Herausforderungen beim klinischen Einsatz von KI bislang kaum fundierte Lösungsansätze dokumentiert sind.

## Fazit für die Praxis

- Instrumentelle Ganganalyse (IGA) ermöglicht eine differenzierte Analyse von komplexen Bewegungsstörungen anhand von hochauflösenden Bewegungsdaten und erhebt Informationen, welche mit konventioneller klinischer Diagnostik nicht erfassbar sind.
- Künstliche Intelligenz (KI) bietet großes Potenzial, um die instrumentelle Ganganalyse von einer expertenbasierten Technologie für ausgewählte klinische Anwendungsfälle zu einer Standarddiagnostik für Bewegungsstörungen weiterzuentwickeln.
- Etablierte KI-Ansätze aus anderen Bereichen sind nur begrenzt auf die Ganganalyse übertragbar.
- Es bedarf der Entwicklung von Leitlinien und Standards für die klinische Anwendung von KI in der Ganganalyse, welche spezifische Lösungsansätze für die wesentlichen ganganalysespezifischen Herausforderungen adressieren.
- Für erfolgreiche ganganalytische KI-Entwicklungen ist die direkte Einbindung von klinischem Expertenwissen unabdingbar.

## Data Availability

Die vorliegende Publikation beinhaltet keine erhobenen Datensätze.

## Abkürzungen

GAMMA: Gesellschaft für die Analyse menschlicher Motorik und ihre klinische Anwendung
IGA: Instrumentelle Ganganalyse
KI: Künstliche Intelligenz
OPS: Operationen- und Prozedurenschlüssel
